# Synergic Involvements of Microorganisms in the Biomedical Increase of Polyphenols and Flavonoids during the Fermentation of Ginger Juice

**DOI:** 10.1155/2020/8417693

**Published:** 2020-08-01

**Authors:** Christian Aimé Kayath, Armel Ibala Zamba, Saturnin Nicaise Mokémiabeka, Meddy Opa-Iloy, Paola Sandra Elenga Wilson, Moïse Doria Kaya-Ongoto, Rodd Jurah Mouellet Maboulou, Etienne Nguimbi

**Affiliations:** ^1^Laboratoire de Biologie Cellulaire et Moléculaire (BCM), Faculté des Sciences et Techniques, Université Marien Ngouabi, BP. 69, Brazzaville, Congo; ^2^Institut National de Recherche en Sciences Exactes et Naturelles (IRSEN), Avenue de l'Auberge Gascogne, BP. 2400, Brazzaville, Congo; ^3^Ecole Nationale Supérieure d'Agronomie et de Foresterie, Université Marien Ngouabi, BP. 69, Brazzaville, Congo

## Abstract

Steered fermentation by microorganisms gives great added value in the nutritional quality of local food. Ginger rhizome naturally contains a myriad of bioactive compounds including polyphenol and flavonoids. The aim of this work was to ferment the ginger juice, to evaluate the biochemical parameters of ginger wine, and to understand the involvement of microorganisms in the bioincrease of polyphenol compounds. Titratable acidity and pH values were determined and showed that pH is around 1.6 at the end of the fermentation when the acidity is around 6.431 g/L. Using colorimetric assay, the total polyphenolic and flavonoid compounds were evaluated throughout the fermentation. The variation of the polyphenol and flavonoid concentrations of the unsweetened sample was around 10.18 to 14.64 mg Eq AG/g and 1.394 to 2.224 mg Eq Cat/g Ms, but those from the sweet sample were around 10.82 to 18.34 mg Eq AG/g Ms and 1.311 to 2.290 mg Eq Cat/g. Using one-step PCR, multiplex techniques with specific primers, with yeast-like phenotype 27.27% (6), have been assigned among 22 isolates to *Saccharomyces cerevisiae.* By using PCR multiplex techniques, *Bacillus licheniformis*, *Bacillus pumilus*, *Bacillus safensis*, and *Saccharomyces cerevisiae* have been identified. Together with *Saccharomyces cerevisiae*, we showed that *Bacillus* sp. are able to secrete enzymatic landscape with some activities up to 50% including cellulase, amylase, pectinase, and protease.

## 1. Introduction

Ginger, from its scientific name *Zingiber officinale* Roscoe, is a plant of the genus *Zingiber* and member of the family Zingiberaceae [1, 2]. This plant, originally from Asia, is currently cultivated in many tropical regions [3]. The rhizome is underground stem used in traditional medicine and in cooking for the preparation of infusions, drinks, and spices. In the Republic of the Congo, ginger is cultivated and consumed in raw form (rhizome), condiments, or as a drink. Ginger juice is among the most locally commercialized juice throughout in Republic of the Congo cities. No published studies have notified that, more and more, the local populations consume a fermented drink based on ginger juice.

The crop of rhizome has been known to harbor multiple virtues such as lowering cholesterol levels and preventing cancer [4]. Ginger rhizomes naturally contain polyphenols which are a group of commonly encountered antioxidants [5]. These compounds of biological interest are characterized by the presence of at least one phenolic motif (aromatic cycle on which one or more –OH groups are grafted). One of the peculiarities of polyphenols lies in their incredible diversity. There are currently more than 8000 phenolic compounds, including 5000 for the subclass of flavonoids. These biomolecules have anticancer and antisclerotic properties [6, 7] and hepatoprotective and antiallergic effects [8, 9]. Several studies have evaluated a myriad of health benefits of ginger in terms of its antidiabetic effect linked to major bioactive constituents [10]. The intracellular localization of the polyphenol compound is well known. It was previously documented that polyphenolic compounds have been found in the vacuole, and a number of them are close to the pectocellulosic wall of plant cells [11]. Many microorganisms like bacteria, fungi, and yeasts are known to secrete enzymes capable of degrading the cell wall and increasing the quantity of released phenolic compounds [12–18].

The choice of rhizome is justified by the fact that ginger juice is the traditional cold drink that is the most consumed in the Republic of the Congo, and ginger contained several bioactive compounds [1, 4–10]. To our knowledge on more cited popular databases, no study has been carried out on attempts to ferment ginger juice by showing the bioincrease of bioactive compounds. We are interested in the involvement of microorganisms in the nutrient values bioincreased during fermentation. The analysis of the microbiological and biochemical quality of this wine will be of major interest for this work.

## 2. Methods

### 2.1. Preparation of Fermented Ginger, Determination of pH, Titratable Acidity, and Alcohol Content of Ginger Wine

The ginger rhizomes were first bought at Brazzaville local markets including Bacongo, Makélékéle, and Bouemba Markets. 80 g of ginger rhizomes was vigorously washed and ground and 1 L of water was added (i.e., 10%). After sifting the mixture, 120 g of sucrose (Nkayi) was added (i.e., 12%). The mixture was distributed in eight containers containing 1 L of the samples dedicated to be tested. The fermentation process of each sample was followed for seven days. The first day has been considered as Day 1 and so on till day 7. Samples were systematically analyzed for biochemical and microbiological qualities. By using HANNA pH-meter HI 99161, pH values of the ginger wine were determined each day by direct measurement. In the side experiments, samples have been performed for the titratable acidity of the ginger wine during fermentation by titrimetry using a 0.05 N sodium hydroxide solution, in the presence of 1% phenolphthalein as an indicator. The calculation of the alcohol rate has been done at the end of fermentation by using seven samples of fermented ginger wine (E1, E2, E3, E4, E5, E6, and E7) at the end of the fermentation (day 7), and this was extended until the twentieth day (day 20). 250 mL of each fermented ginger wine samples was measured, poured into a 1 L flask, and then connected to the refrigerant. The fermented drink was heated to the boiling point. Distillation is stopped after collecting more than 3/4 of the volume of the test tube. For this, heating is stopped, the flask is cooled to remove the remaining wine, and the distillate is then brought to room temperature of the wine. The percentage of alcohol is evaluated at 20°C by using an alcoholometer.

### 2.2. Extraction of Phenolic Compounds

#### 2.2.1. Drying of Ginger Roots and Fermentation

The ginger roots were washed thoroughly with water and cut into thin rings. After having ground these rings with the aid of a mortar, the grounded product obtained was dried in an oven set at 50°C for 72 hours. The extracts to be used for the determination of total polyphenols were obtained by mixing 50 g of the dried plant material in 500 mL (10%) of distilled water in Erlenmeyer flask. The mixtures are stirred for 72 hours and then filtered. The filtrate obtained is distributed in six small vials due to 30 mL. Another part of the filtrate is chaptalized by adding 20 g of the sugar (SARIS, Nkayi) (4%) until complete dissolution. The samples are analyzed at T0 (first day), T1 (4 days of fermentation), T2 (8 days of fermentation), T3 (12 days of fermentation), and T4 (16 days of fermentation). The contents of vials T0 to T4 have been kept at shelter from light waiting to be analyzed.

#### 2.2.2. Determination of Total Polyphenols

The reagent used is the “Folin–Ciocalteu” reagent. The total polyphenolic compounds are determined in the following manner. 0.1 mL of the plant extract is introduced into Eppendorf tube, the extract is then diluted with 0.9 mL of distilled water, then 0.9 mL of the Folin–Ciocalteu reagent (1 N) is added, and then immediately, 0.2 mL of Na_2_CO_3_ solution (20%) is added. The mixture obtained is incubated at room temperature for about 40 minutes, protected from light. The absorbance is then measured using a spectrophotometer at 725 nm with a methanol solution used as a blank. Calibration line is previously carried out before analysis with gallic acid under the same conditions as the samples to be analyzed. The results obtained are expressed in mg gallic acid equivalent per gram of dry matter (E AG/g Ms).

#### 2.2.3. Determination of Total Flavonoids

The reagents used consist of the colorless solution of sodium nitrite (NaNO_2_, 5%) and aluminum chloride (AlCl_3_, 10%). The total flavonoids are evaluated by colorimetry; 250 *μ*L of the extract and 1 mL of distilled water are successively introduced into a 10 ml flask. At the initial time (0 minutes), 75 *μ*L of a solution of NaNO_2_ (5%) is added; after 5 minutes, 75 *μ*L of AlCl_3_ (10%) is added. And after 6 minutes, 500 *μ*L of NaOH (1 N) is added and 2.5 mL of distilled water is added successively to the mixture. A calibration curve is drawn up with standard solutions of catechin prepared at different concentrations. The absorbance of the mixture obtained is directly measured with a UV-visible spectrophotometer at 510 nm, and the results are expressed in mg equivalent catechin/gr of dry matter (EC/g Ms).

### 2.3. Isolation of Microorganisms

At the end of 7 days of fermentation, the ginger wine was collected to make microbiology assessment. Dilutions were done, and microorganism suspension was streaked on Sabouraud supplemented with chloramphenicol, 100 *μ*g/mL, and Mossel supplemented with polymyxin B. Enumeration of colonies was done in triplicate on plate count agar (PCA). The Petri dishes were incubated at 37°C for 24 h to 48 h. After the first isolation on Petri dishes, each colony of different appearance was separately isolated. Purification of the isolates was rigorously done by successive and alternating subcultures. Purity was estimated by using a microscope for morphological characterization. Gram status was determined by using 3% KOH. Sporulation, hydrogen peroxide (H_2_O_2_), and oxidases tests were used for biochemical characterization.

### 2.4. Determination of Proteolytic Activity

Some *Bacillus* strains were assessed for the ability to secrete proteases in the extracellular environment as described and modified by Kaya-Ongoto et al. [19]. Briefly, 1 g of agarose was weighed and mixed with 100 mL of PBS. The mixture was heated in a microwave for 3 min until agarose was completely dissolved and then cooled in a water bath at 40°C. Then, 10 mL of skim milk was added to the mixture. After homogenization, the mixture was poured into the Petri dishes. Once solidified, wells were carefully and aseptically generated into the gels. A volume of 50 *μ*L of the overnight culture supernatant is deposited in the wells made on the agar medium composed of 1% agarose gel, 0.01 M PBS, pH 7.4, and skimmed milk. The Petri dishes are incubated at 37°C for 24 hours. The presence of proteolytic activity is detected by a clear halo around colonies indicating hydrolysis of casein. The halo diameters were measured.

### 2.5. Amylolytic, Cellulolytic, and Pectinolytic Activities

To assess the amylolytic, cellulolytic, and pectinolytic activities, an overnight culture was carried out on Petri dishes containing LB medium for the purpose of well-isolated colonies. Then, a young bacterial colony was deposited on the surface on solid LB medium separately added to 1% of starch, 0.5% of cellulose, and 0.5% of pectin. The Petri dishes are incubated 48 h to 72 h. The revelation is made with Lugol. A test is positive if there is a clear halo around the colony corresponding to a lysis range. The percentage of enzymatic activity was determined according to the following formula: % = DT-DC/DT with DT: total lysis diameter (lysis area + colony diameter); DC: colony diameter; and %: percentage of lysis.

### 2.6. Molecular Identification of Microorganisms

The recent molecular identification using the *fibE* gene encoding for the fibrinolytic enzyme has been used for targeting strains like *Bacillus amyloliquefaciens*, *B. subtilis*, *B. pumilus*, *B. licheniformis*, *B. altitudinis*, *B. mojavensis*, *B. safensis*, and *B. atrophaeus*. Yeast has been identified by using the molecular methods. Primers used in this work are indicated in Table 1 with the corresponding targeted microorganisms. Extraction and purification of isolate genomic DNA were performed according to the NucleoSpin Microbial DNA (Macherey-NAGEL) kit. Briefly, isolates were grown in 5 mL LB broth for 24 h at 37°C with stirring. The DNA purity was assessed by electrophoresis on agarose gel and by the ratio of optical densities 260/280 nm. 5 *μ*L of each amplification product was mixed with 2 *μ*L of loading buffer (BIOKE). Mixtures were subjected to electrophoresis on 1% agarose gel (w/v). The 10 kb 2-Log (BIOKE) was used as a molecular weight marker.

## 3. Statistical Analysis

Principal component analysis (PCA) was used to investigate possible correlations between isolates and enzymatic activity. Prior to ordination, percentage of enzymatic activity data was transformed to better meet the assumptions of normality [21] using ln (*x* + 1). All analyses were performed using CANOCO (Canonical Community Ordination, version 4.5) [22].

## 4. Results

### 4.1. Obtaining the Ginger Wine Fermentation Diagram

**Figure 1:**
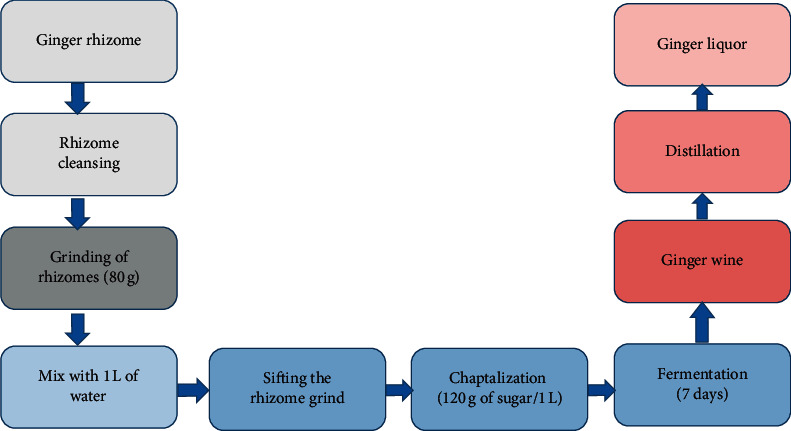
The lab process of fermentation of ginger wine.

Ginger wine is not a local drink. We tried to make this drink by performing the chaptalization technique of adding sugar with ginger juice.  Figure 1 shows us the manufacturing diagram for the lab fermentation of ginger juice.

### 4.2. Evaluation of Biochemical Parameters

**Figure 2:**
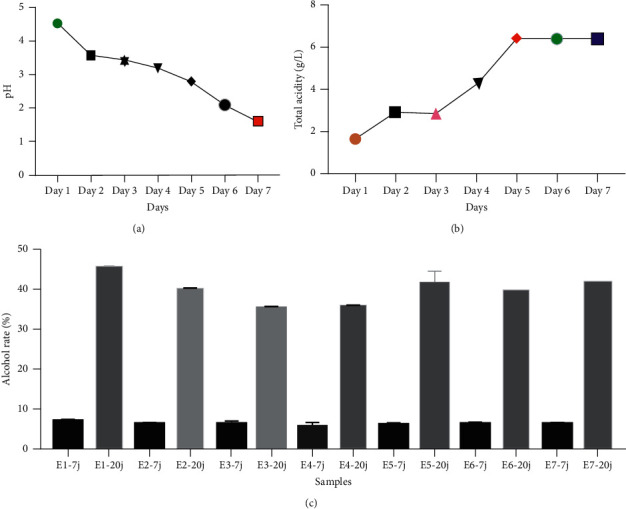
Determination of pH, titratable acidity, and alcohol content of ginger wine. (a) Evaluation of pH during fermentation of ginger. (b) Titratable acidity: days 1, 2, 3, 4, 5, 6, and 7. (c) Alcohol content of different samples. E1, 2, 3, 4, 5, and 7-7j: samples collected after the seventh day. E1, 2, 3, 4, 5, and 7-20j: samples collected after the 20^th^ day.

The measurements of the pH values obtained from the 1^st^ day of fermentation until the 7^th^ of fermentation are presented (Figure 2). After sieving and just the first day of fermentation, pH of ginger is around 4.5 and decreases considerably at the end of fermentation to 1.6 (Figure 2(a)). As explained in the methods, the ginger wine was distilled after seven days; the alcohol level was around 7%. To optimize the alcohol level, the fermentation was extended until the twentieth day. The results show a clear increase in the alcohol rate up to 45% (Figure 2(c)). In order to know the type of fermentation of ginger juice, we carried out titratable acidity. The values are between 1.687 g/L and 6.421 g/L (Figure 2(b)).

### 4.3. Determination of Total Polyphenols and Flavonoids

**Figure 3:**
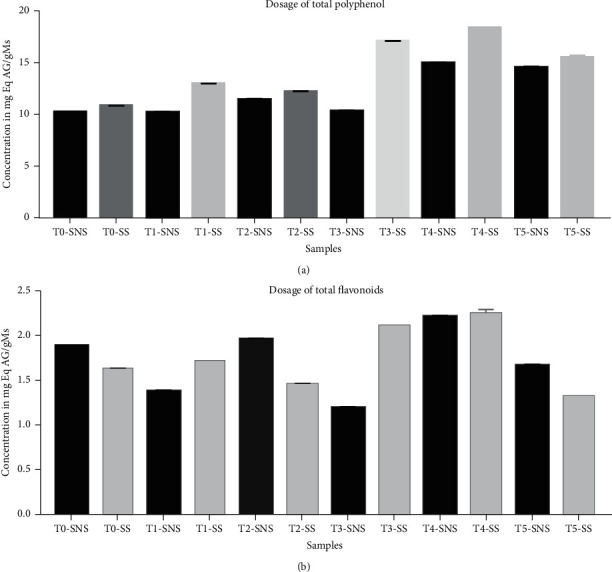
Bioincrease of polyphenol (a) and flavonoids (b) during the fermentation of ginger juice from a sweet sample (SS) and nonsugar added sample (SNS). T0: first day, T1: fourth day after fermentation, T2: eighth day after fermentation, T3: twelfth day after fermentation, T4: sixteenth day after fermentation, and T5: twentieth day after fermentation.

Polyphenols constitute a family of organic molecules widely present in the plant kingdom. They are characterized, as the name suggests, by the presence of at least two phenolic groups associated in more or less complex structures, generally of high molecular weight. In order to quantify these molecules, we carried out as mentioned in the methods. As results, we showed that for the unsweetened sample, the polyphenol concentration varies from 10.18 to 14.9 mg Eq AG/g Ms after 16 days of fermentation (Figure 3(a)). As for the sweet sample, the polyphenol concentration ranges from 10.82 to 18.3 mg Eq AG/g Ms after 20 days of fermentation (Figure 3(a)). It can be seen that the polyphenol concentrations in the sweet sample are higher than those in the unsweetened sample (Figure 3). For the unsweetened sample, the flavonoid concentration varies from 1.39 to 2.22 mg Eq Cat/g Ms after 16 days of fermentation (Figure 3(b)). After 16 days of fermentation, there is an increase in the concentration, i.e., 2.224 mg Eq Cat/g Ms (FIG. 9). For the sweet sample, the flavonoid concentration varies from 1.31 to 2.39 mg Eq Cat/g Ms (Figure 3(b)).

### 4.4. Enzymatic Activities in the Bioincrease of Antioxidants

**Figure 4:**
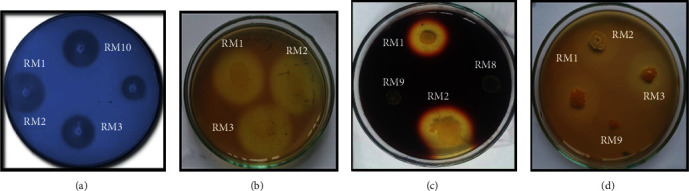
Illustration of some enzymatic activities carried out on isolates from ginger wine. (a) Proteolytic activity. (b) Cellulolytic activity. (c) Amylolytic activity. (d) Pectinolytic activity. RM1, 2, 3, 8, 9, and 10: isolates.

The ability isolates suspected like *Bacillus* to secrete several exocellular enzymes and to coordinate the action with the increase of antioxidants in the fermentation process of ginger juice allow us to find out for enzymatic activities. Isolates have been tested for their ability to degrade milk casein, cellulose, starch, and pectin. As explained in the methods, the profiles of different enzymatic tests carried out are illustrated in Figure 4.

**Figure 5:**
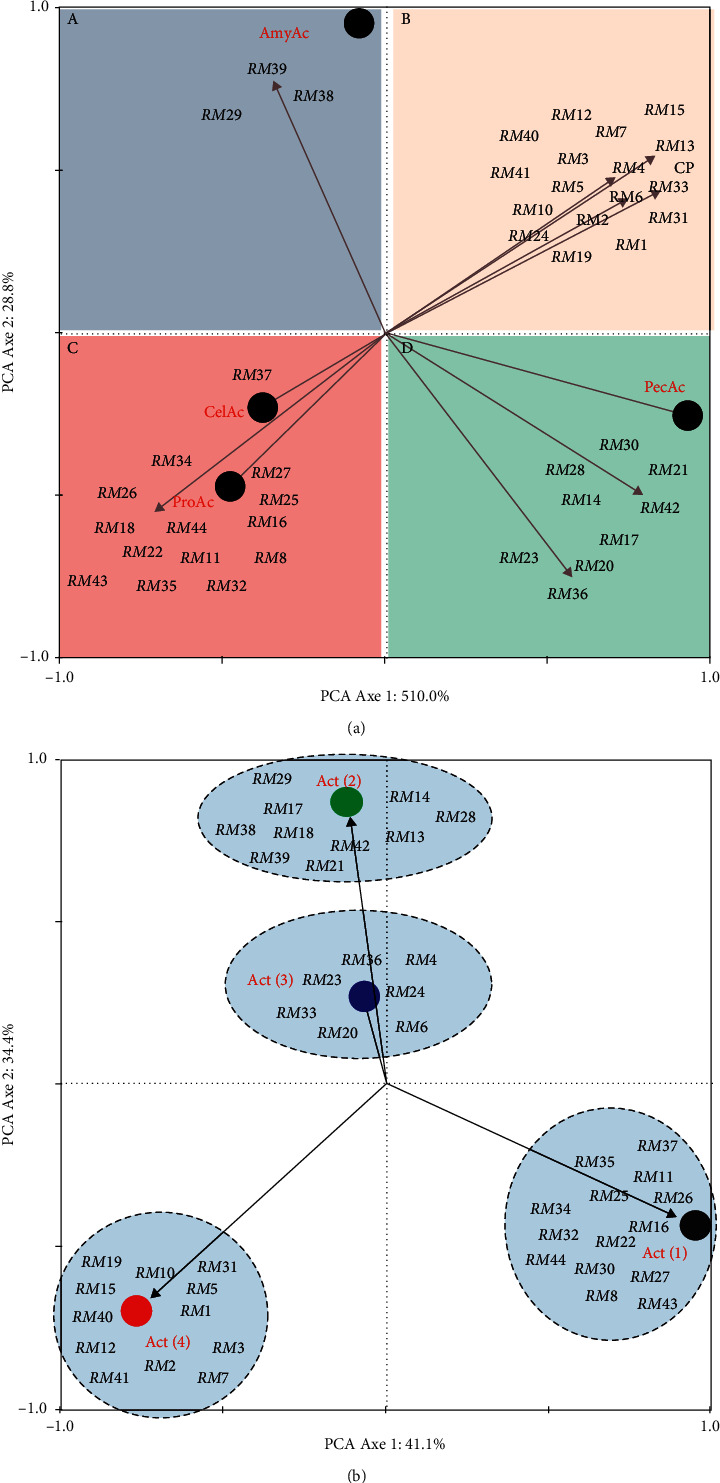
(a) PCA of isolates with one enzymatic activity: proteolytic (ProAc), cellulolytic (CelAc), amylolytic (AmyAc), and pectinolytic (PecAc). (b) PCA of isolates with one enzymatic activity (Act1), 2 enzymatic activities (Act2), 3 enzymatic activities (Act3), and 4 enzymatic activities (Act4): PCA: principal component analysis. RM1 to 44: isolates 1 to 44.

The halo diameters were evaluated in centimeters (cm) after 24 hours of incubation at 37°C. A total of forty isolates tested positive for the proteolytic test; eighteen of the forty-four isolates exhibited cellulolytic activity with varying diameters. Twenty and twenty-five out of forty-four isolates tested were positive for the amylolytic and pectinolytic activities after 24 h, respectively. In dial (a), the isolates only developed amylolytic activity (RM29, 38, and 39); in dial (b), the isolates developed all amylolytic, cellulolytic, pectinolytic, and proteolytic activities. In dial (c), we note the isolates have the cellulolytic (RM37) and proteolytic (RM8, 11, 16, 18, 22, 25, 26, 27, 32, 34, 35, 43, and 44) activities. Finally, in dial (d), bacterial isolates have developed only pectinolytic activity (RM14, 17, 20, 21, 23, 28, 30, 36, and 42) (Figure 5(a)). 31,81% (14) have developed a single activity either proteolytic or amylolytic or cellulolytic and either pectinolytic. RM8, 11, 16, 22, 25, 26, 27, 30, 32, 34, 35, 37, 43, and 44 are part of this group. 22.72% (10) presented two activities at the same time (cellulolytic activity and pectinolytic-amylolytic, cellulolytic-proteolytic activity) which includes isolates RM14, 13, 17, 18, 39, 21, 28, 29, 38, and 42. 15.90% (7) have developed three activities (RM4, 6, 20, 23, 24, and 33) and 27.27% (13) have finally presented four activities at the same time. This includes RM1, 2, 3, 5, 7, 10, 12, 15, 19, 31, 40, and 41. RM9 and RM30 are not able to develop any enzymatic activity (Figure 5(b)).

**Figure 6:**
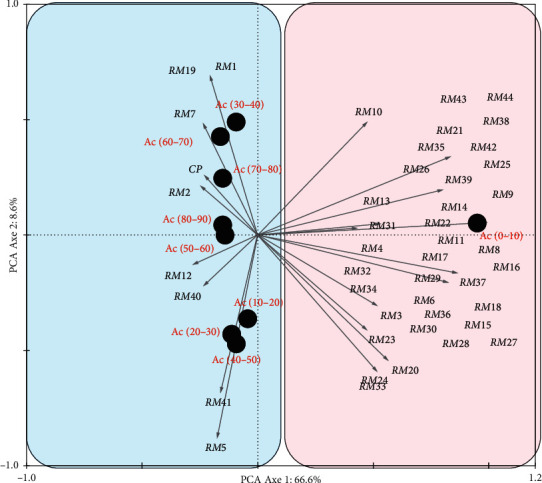
PCA of isolates with interesting percentage of enzymatic activities: proteolytic, cellulolytic, amylolytic, and pectinolytic. PCA: principal component analysis. RM1 to 44: isolates 1 to 44.

With the exception of RM4, 9, 13, 30, and 37, all the isolates are capable of degrading the casein used as a substrate by the proteases secreted by bacteria of the bacillus genus. However, very variable percentages have been observed with regard to the ability to degrade cellulose, pectin, and amylose. RM1, 5, 7, 12, 19, 40, and 41 are part of those where the percentages are up to 30%. Other isolates have percentages between 0 and 10% (Figure 6).

### 4.5. Microbiological Landscape and Molecular Identification

**Figure 7:**
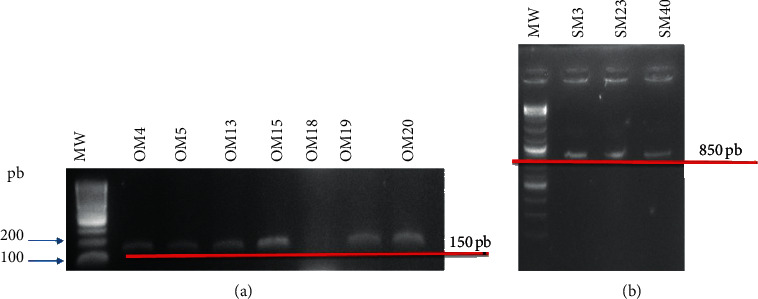
(a) 1% agarose gel electrophoresis after amplification using 2 pairs of oligonucleotides of the isolates (OM4, OM5, OM13, OM15, OM17, OM18, OM19, and OM20). MW: molecular weight. (b) The electrophoretic profile on agarose gel (1%) obtained using the fibE-specific primers to each species of *Bacillus*: SM3, S23, and SM40.

By using Sabouraud and Mossel, 22 isolates with yeast orientation and 44 isolates with *Bacillus* orientation have been obtained. Among 22 yeast-oriented isolates, we used specific oligonucleotides for six (6) *Saccharomyces* sp. including *Saccharomyces cerevisiae*, *S. mikatae*, *S. paradoxus*, *S*. *arboricolus*, *S*. *kudriavzevii*, and *S. bayanus*. Agarose gel electrophoresis revealed a single band at 150 *bp* corresponding to the isolates OM4, OM5, OM13, OM15, OM19, and OM20. All isolates were assigned to *S. cerevisiae* (Figure 7(a)). Other isolates have not been identified based on the targeted species (Figure 7(a)).

Isolates with good profiles based on enzyme activities were selected for the extraction of genomic DNA. A total of seven isolates were selected: RM1, 3, 5, 7, 12, 19, 23, 40, and 41. The revelation was made on 1% agarose gel by using BET. After the amplification using the specific primers, the results show that only three pairs of specific primers allowed the amplification of the fibE gene, in particular the couple fibE-Bp, fibE-Bl, and fibE-Bsa, primers targeting, respectively, *B. pumilus*, *B. licheniformis,* and *B. safensis* (Figure 7(b)).

## 5. Discussion

The aim of this work was to understand the involvement of microorganisms in the bioincrease of phenolic compounds during the fermentation of ginger juice. In this way, we have first of all made the ginger wine and, as a result of biochemical analysis, the alcoholic degree after distillation is between 35 and 45%. These high levels of alcohol could be explained by the presence of microorganisms such as yeasts [23] promoting with chaptalization which is an additional advantage of microorganisms. Titratable acidity is a measure of the total acid concentration. In the titration with a base, all the H+ ions are neutralized whether they are ionized or not. We have also shown that titratable acidity increases during fermentation. It turns out that the average acidity ranges between 1.687 and 6.431 g/L. This acidity is caused by the fermentation activity of microorganisms which degrade cell wall by releasing some organic acids in the medium [24]. The pH has shown values down to 1.6 ± 0.1. Some fermented drinks have been shown with values around 4.5 ± 0.2. The type of fermentation assigned to pineapple wine is purely lactic fermentation. Ginger rhizomes are rich in omega 3 and 9 fatty acids (oleic and linolenic acids) [5]. The coordinated action of enzymes secreted by yeasts and bacteria of the *Bacillus* genus would help acidify ginger wine. The pH indicates the acidity and the alkalinity of a product while the titratable acidity indicates the quantity of acids present in the ginger juice. The pH and titratable acidity of the ginger juice were used to estimate the consumability and properties that are not visible. Both criteria can be considered as indicators of organoleptic characteristics. Acids play an important role in the quality of modern or traditional wines because the flavor is essentially and indisputably a balance between the sugar and acid content.

In this work, we have shown that polyphenols and flavonoids increase during fermentation of ginger. Microorganisms are strongly implicated in this process [14, 16, 17, 25]. Using molecular biology techniques, we have shown *Saccharomyces cerevisiae* is mainly isolated from ginger wine as well as bacteria of the genus *Bacillus* like *B. licheniformis*, *B. safensis*, and *Bacillus pumilus*. Together with *Saccharomyces cerevisiae*, they play a primordial role in the bioincrease of the production of polyphenols and flavonoids [26]. *Saccharomyces cerevisiae* has been shown to produce *β*-glucosidase and feruloyl esterase for cell wall degradation [18, 27]. The *fibE* gene encoding a fibrinolytic enzyme was used as a biomarker to identify isolates. This method has an advantage of being reliable and rapid and has great discriminatory power as shown by Kaya-Ongoto et al. [19]. In total, three strains of *Bacillus* including B. *licheniformis, B. pumilus*, and *B. safensis* belonging to phylogenic group I were identified. These results are consistent with previous works showing that bacteria of the genus *Bacillus* can be isolated from fermented foods [19, 28–31].

The total polyphenol concentrations of the unsweetened sample vary from 10.18 to 14.64 mg Eq AG/g Ms. As fa as the sweet sample is concerned, values vary from 10.82 to 18.34 mg Eq AG/g Ms. The flavonoid concentrations in the unsweetened sample range from 1.394 to 2.224 mg Eq Cat/g. The flavonoid concentrations in the sweet sample vary from 1.311 to 2.290 mg Eq Cat/g. It is possible to accept that chaptalization is an important factor for promoting the growth of yeasts. The high density of microorganisms linked to the secretion of pectinolytic, amylolytic, and cellulolytic enzymes explains this increase. These enzymes are directly responsible for the destruction of the cell walls of plant cells, thereby causing this increase [26]. However, we note that in the nonchaptalized samples (no added sugar), there is also an increase in total phenolic compound and total flavonoids as well. This increase is explained by the composition of crop that contains sucrose, glucose, and fructose [32, 33] which allow microorganisms to grow and to take a benefit in the environment carbon. Soluble and insoluble carbohydrates are the most important components in crop rhizome and contribute to their nutritive value [33]. Given the importance of polyphenols as antioxidants, one could safely say that the fermentation of ginger juice offers the possibility of studying polyphenols and extracting them. This first study needs to be deepened by targeting other types of polyphenols useful to contribute to the metabolic pathways for good health. The decrease in total polyphenols and flavonoids after the seventh day can be explained by the fact that these compounds can also become potential sources of carbon of microorganisms. Interactional synergies of *Saccharomyces cerevisiae* and *Bacillus* sp., the emergence of new microorganism groups during fermentation could impact the increase and/or decrease in total polyphenols.

The genus *Bacillus* is known for its ability to produce extracellular enzymes such as amylases [34], pectinases [35–37], cellulases [18, 38, 39], proteases [40, 41], and other biomolecules as well [42]. In the context of this work, the results are promising because some identified isolates and nonidentified ones have shown degradation percentages of cellulose, amylose, and pectin up to 30% depending on the activity (cellulolytic, pectinolytic, or amylolytic) and also lysis diameters of the order of 2.2 cm ± 0.03 in the case of proteolytic activity. All the enzymatic arsenal could thus make it possible to degrade the wall of the plant cell by allowing the release of biomolecules like the antioxidants, being inside the plant cells of the ginger rhizomes towards the outside. The enzymatic activities carried out have shown that bacteria of the genus *Bacillus* secrete lytic enzymes produced during fermentation that could contribute to the degradation of cell wall structures resulting in biomolecular release such as polyphenols and flavonoids. This therefore optimizes the amount of polyphenols in this drink [43]. Patients suffering from diabetes mellitus [44] and obesity [45, 46] are not obliged to chaptalize ginger juice in order to have a benefit from the polyphenols released during the fermentation orchestrated by microorganisms. In this work, we have shown that nonchaptalized ginger juice can also be bioincreased. Indeed, microorganisms like *Saccharomyces* sp. and *Bacillus* sp. isolated from ginger wine are also able to use the sugars of rhizomes and many other molecules [5] by releasing the enzymes which will destroy the walls of plant cells.

Several studies reported that fermentation influences the polyphenolic profile of extracts obtained from various plant sources or during the fermentation of plant sources. This concerns *Bacillus pumilus* and *Bacillus subtilis* on the fermentation of soybean and cheonggukjang [16, 47]; *Lactobacillus acidophilus* on apple juice; *Lactobacillus plantarum*, *Lactobacillus acidophilus*, *Lactobacillus johnsonii*, *Lactobacillus reuteri*, *Lactobacillus acidophilus*, and *Lactobacillus delbrueckii* on whole grain barley, oat groat, and soybean [14, 48]; and *Saccharomyces cerevisiae* on wheat bran [17]. In this work, we demonstrate that *Saccharomyces cerevisiae*, *Bacillus licheniformis*, *Bacillus pumilus*, and *Bacillus safensis* can easily and synergically play this role by using both genera enzymatic landscapes that can degrade plant material.

## 6. Conclusion

The consumption of rhizomes or ginger juice is a real health ally. The nutritional quality of people with diabetes remains a real challenge in the Republic of the Congo. In this work, we have shown that the fermentation process of ginger juice is necessary to obtain the high values of antioxidants like polyphenols and flavonoids. People with diabetes and obesity can also consume ginger without the need to add sugar. Microorganisms like *Saccharomyces cerevisiae*, *Bacillus pumilus, Bacillus licheniformis*, and *Bacillus safensis* secrete enzymes which are able to bioincrease polyphenols and especially flavonoids during the fermentation of ginger juice. More studies are on the way to optimize other bioactive substances and/or fermentation conditions in Congo ginger wine.

## Figures and Tables

**Table 1:** Primers used in this work.

Oligo names	5'----3'	Size (pb)	Targeted species	References
*For group 1 Bacillus identification*				
Ba.IdMa-F	GCGCAGTCCGTGCCTTACGGCGT	828	*B. amyloliquefaciens*	[19]
Ba.IdMa-R	TTACTGAGCTGCCGCCTGTACG			
Bl.Id.Ma-F	GCGCAAACCGTTCCTTACGGCAT	825	*B. licheniformis*	
Bl.Id.Ma-R	TTATTGAGCGGCAGCTTCGAC			
Bs.Id.Ma-F	GCGCAATCTGTTCCTTATGGCAT	835	*B. subtilis*	
Bs.Id.Ma-R	TTATTGTGCAGCTGCTTGTACGTTGA			
Bp.Id.Ma-F	GCACAAACCGTCCCTTATGGAAT	828	*B. pumilus*	
Bp.Id.Ma-R	TTAGTTAGAAGCCGCTTGAGCG			
Bm.Id.Ma-F	GCGCAATCTGTTCCTTACGGCAT	837	*B. mojavensis*	
Bm.Id.Ma-R	TTATTGTGCAGCTGCCTGCAC			
Bsa.Id.Ma-F	GCACAAACCGTCCCTTATGGAAT	828	*B. safensis*	
Bsa.Id.Ma-R	TTAGTTAGAAGCCGCTTGAACGTTG			
Bat.Id.Ma-F	GCTCAGTCA GTACCTTATG GCAT	828	*B. atrophaeus*	
Bat.Id.Ma-R	TTATTGCGCTGCTGCCTGAACG			
Bal.Id.Ma-F	GGTCAAAGCGTCCCTTATGGTA	828	*B. altitudinis*	
Bal.Id.Ma-R	TTATCGTGCAGCTTTTTGTAC			

*For yeast identification*				
Sbay F1	GCTGACTGCTGCTGCTGCCCCCG	275	*Saccharomyces bayanus*	[20]
Sbay R1	TGTTATGAGTACTTGGTTTGTCG			
Scer F2	GCGCTTTACATTCAGATCCCGAG	150	*Saccharomyces cerevisiae*	
Scer R2	TAAGTTGGTTGTCAGCAAGATTG			
Sarb_F1	GGCACGCCCTTACAGCAGCAA	349	*Saccharomyces arboricolus*	
Sarb_R2	TCGTCGTACAGATGCTGGTAGGGC			
Skud_F2	ATCTATAACAAACCGCCAAGGGAG	660	*Saccharomyces kudriavzevii*	
Skud_R1	CGTAACCTACCTATATGAGGGCCT			
Smik_F1	ACAAGCAATTGATTTGAGGAAAAG	508	*Saccharomyces mikatae*	
Smik_R1	CCAGTCTTCTTTGTCAACGTTG			
Spar_F7	CTTTCTACCCCTTCTCCATGTTGG	739	*Saccharomyces paradoxus*	
Spar_R7	CAATTTCAGGGCGTTGTCCAACAG			

## Data Availability

The Excel sheet including the data used to support the findings of this study is available from the corresponding author upon request.
